# Cardiac, bone and growth plate manifestations in hypocalcemic infants: revealing the hidden body of the vitamin D deficiency iceberg

**DOI:** 10.1186/s12887-018-1159-y

**Published:** 2018-06-26

**Authors:** Suma Uday, Nadja Fratzl-Zelman, Paul Roschger, Klaus Klaushofer, Ashish Chikermane, Vrinda Saraff, Ted Tulchinsky, Tom D. Thacher, Tamas Marton, Wolfgang Högler

**Affiliations:** 1 Department of Endocrinology & Diabetes, Birmingham Women’s and Children’s Hospital, Steelhouse Lane, Birmingham, B4 6NH UK; 2 Institute of Metabolism and Systems Research, University of Birmingham, Birmingham, UK; 3 1st Medical Department Ludwig Boltzmann Institute of Osteology at Hanusch Hospital of WGKK and AUVA Trauma Centre, Meidling, Vienna, Austria; 4 Department of Cardiology, Birmingham Women’s and Children’s Hospital, Birmingham, UK; 5 Emeritus, Braun School of Public Health and Community Medicine, Hadassah Medical Center, Hebrew University-Hadassah, Ein Karem, Jerusalem, Israel; 6 Department of Family Medicine, Mayo Clinic, Rochester, MN USA; 7 Department of Cellular Pathology, Birmingham Women’s and Children’s Hospital, Birmingham, UK

**Keywords:** Rickets, Hypocalcemia, Cardiomyopathy, Seizures, Policy, Vitamin D

## Abstract

**Background:**

Whilst hypocalcemic complications from vitamin D deficiency are considered rare in high-income countries, they are highly prevalent among Black, Asian and Minority Ethnic (BAME) group with darker skin. To date, the extent of osteomalacia in such infants and their family members is unknown. Our aim was to investigate clinical, cardiac and bone histomorphometric characteristics, bone matrix mineralization in affected infants and to test family members for biochemical evidence of osteomalacia.

**Case presentation:**

Three infants of BAME origin (aged 5–6 months) presented acutely in early-spring with cardiac arrest, respiratory arrest following seizure or severe respiratory distress, with profound hypocalcemia (serum calcium 1.22–1.96 mmol/L). All infants had dark skin and vitamin D supplementation had not been addressed during child surveillance visits. All three had severely dilated left ventricles (z-scores + 4.6 to + 6.5) with reduced ejection fraction (25–30%; normal 55–70), fractional shortening (7 to 15%; normal 29–40) and global hypokinesia, confirming hypocalcemic dilated cardiomyopathy. They all had low serum levels of 25 hydroxyvitamin D (25OHD < 15 nmol/L), and elevated parathyroid hormone (PTH; 219–482 ng/L) and alkaline phosphatase (ALP; 802–1123 IU/L), with undiagnosed rickets on radiographs.

One infant died from cardiac arrest. At post-mortem examination, his growth plate showed a widened, irregular zone of hypertrophic chondrocytes. Histomorphometry and backscattered electron microscopy of a trans-iliac bone biopsy sample revealed increased osteoid thickness (+ 262% of normal) and osteoid volume/bone volume (+ 1573%), and extremely low bone mineralization density. Five of the nine tested family members had vitamin D deficiency (25OHD < 30 nmol/L), three had insufficiency (< 50 nmol/L) and 6/9 members had elevated PTH and ALP levels.

**Conclusions:**

The severe, hidden, cardiac and bone pathology described here exposes a failure of public health prevention programs, as complications from vitamin D deficiency are entirely preventable by routine supplementation. The family investigations demonstrate widespread deficiency and undiagnosed osteomalacia in ethnic risk groups and call for protective legislation.

## Background

Dark skin pigmentation, lack of sunshine and extensive clothing reduce cutaneous vitamin D production, increasing the risk of hypocalcemia, rickets, and osteomalacia. Traditional diets low in calcium impose the same risk and exacerbate the effect of vitamin D deficiency [1, 2]. Hence, rickets and osteomalacia are a major public health concern in South Asia, Africa and the Middle East. The last century has witnessed a global migration from these regions to high-income nations, resulting in changes in population demographics and new public health challenges. Most high-income countries are geographically located in latitudes whose seasonally absent ultraviolet sunlight spectrum reduces vitamin D status. Whilst rickets is considered a rare disease in high-income countries it is highly prevalent among Black, Asian and Minority Ethnic (BAME) group with darker skin [3, 4]. Amongst those ethnic risk groups live the most vulnerable subgroup with no voice – infants. The United Kingdom has the lowest adherence to infant vitamin D supplementation in Europe [5] and hypocalcemic seizures, heart failure and rickets occur nearly exclusively in BAME group [5–8]. To date, there are no bone biopsy or biochemical data on the extent of disease of undiagnosed osteomalacia in affected infants and their families.

## Case presentation

Here we present 3 infants, all born in England to mothers of BAME origin, with serious complications from vitamin D deficiency (serum 25-hydroxyvitamin D [25OHD] concentration < 30 nmol/L) presenting in early-spring, and biochemical investigations of their family members. All three infants had hypocalcemic dilated cardiomyopathy and hidden rickets, of whom one died following cardiac arrest and whose post-mortem bone ultrastructural analysis revealed severe, undiagnosed bone and growth plate pathology.

### Clinical, cardiac, laboratory and radiological characteristics

Clinical, anthropometric, laboratory, electro- and echocardiography data were extracted from medical notes. X-rays were taken as part of routine clinical care or post-mortem. Blood samples of patients, siblings and parents were analysed for serum calcium, phosphate, alkaline phosphatase (ALP), 25OHD and parathyroid hormone (PTH) using routine laboratory methods. Specific reference was made to information provided to the family at birth and adherence to child surveillance visits.

### Bone and growth plate histology and backscattered electron microscopy

Bone samples taken during routine post-mortem of patient 1 were processed as follows: A 7th rib growth plate section was assessed using Elastica van Gieson staining. A trans-iliac bone biopsy sample was taken and histomorphometric analyses were performed using standard procedures [9]. Bone mineralization density distribution (BMDD), reflecting the calcium content of bone matrix, was measured by quantitative backscattered electron microscopy as described previously [10]. The BMDD curve of patient 1 was compared with a young reference population [10]. Parents of all 3 patients provided informed consent for publication.

#### Patient 1

**Fig. 1:**
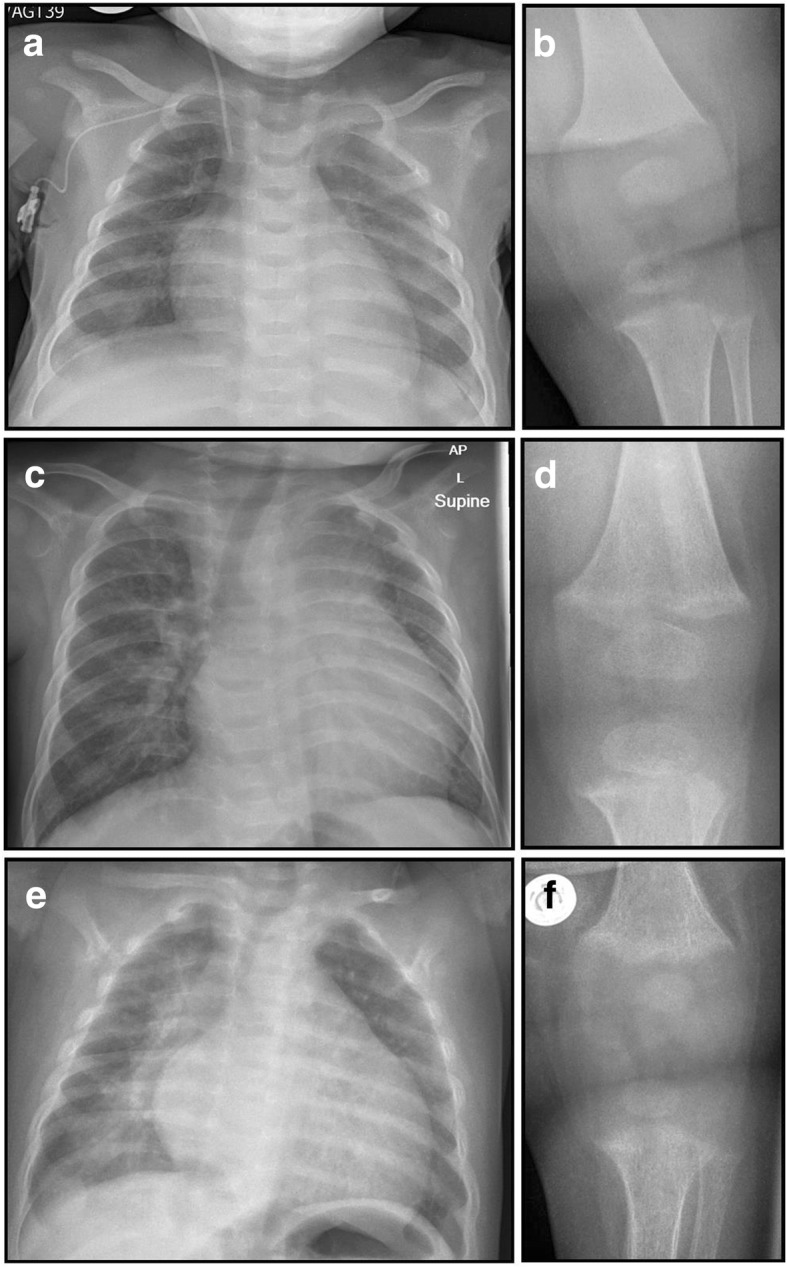
Radiographs. Chest and knee radiographs of Patient 1 (**a, b**), 2 (**c, d**) and 3 (**e, f**) demonstrate cardiomegaly and rickets

A 6-month old exclusively breastfed, African boy presented to the emergency department (ED) with an out-of-hospital cardiac arrest. In the weeks prior to presentation, he had 3 brief episodes of peri-oral cyanosis and pallor and presented twice to ED with increased work of breathing. On initial assessment by paramedics he showed no signs of life and was in asystole. He was resuscitated until spontaneous circulation was restored at 36 min. Investigations revealed low ionised calcium (0.72 mmol/L), warranting repeated intravenous calcium boluses followed by continuous infusion. Cefotaxime was commenced for presumed sepsis, and oseltamivir was added after isolating influenza A on a nasal swab. Intravenous fluids and inotropes were administered. In the intensive care unit, an echocardiogram showed severe dilated cardiomyopathy with poor left ventricular ejection fraction (LVEF) of 25–30% [normal 55–70%]), fractional shortening (FS) of 7% [normal 29–40%], dyskinetic septal motion, global hypokinesia, and moderate to severe mitral regurgitation with a structurally normal heart. Rickets was confirmed radiographically (Fig. 1b), with elevated serum ALP and PTH concentrations, and low 25OHD < 15 nmol/L (Table 1). Cholecalciferol (6000 IU daily) was commenced, and intravenous calcium was continued until serum calcium normalised (72 h). Cardiac failure was managed with diuretics and vasodilators. Brain Magnetic resonance imaging (MRI) revealed severe hypoxic-ischaemic encephalopathy, correlating with the clinical finding of unresponsiveness to external stimuli. The care team and family elected to withdraw life support, and the infant died 6 days after presentation.

**Table 1:** Characteristics of the three cases at presentation

	Patient 1	Patient 2	Patient 3
Presentation & Demographics			
Age	6 months	6 months	5 months
Ethnicity	South African-Ghanaian	Somali	British Pakistani
Month of presentation	January	February	February
Presenting feature	Cardiac arrest at home; down time of 36 min	Respiratory arrest and seizure; apnoea (~ 4 min)	Cough, difficulty breathing and poor feeding
Feeding mode at presentation	Exclusively breastfed	Breastfed (solids started 2 weeks earlier)	Exclusively breastfed
Birth weight in kg (centile)	3.7 (91st)	4.0 (98th)	1.75 at 35 weeks (9th)
Development	Normal	Normal	Normal
Immunisation	Up to date	Up to date	Up to date
Length cm (centile)	68 (50th)	71 (91st)	58 (0.4th)
Weight kg (centile)	8 (50th)	8.5 (91st)	4.5 (< 0.4th)
Investigations			
Adjusted serum Calcium (2.2–2.7 mmol/L)^b^	1.60	1.22	1.96
PO_4_ (1.3–2.4 mmol/L)	0.47^a^	1.95	0.69
ALP (105–420 IU/L)	802	996	1391
PTH (12–29 ng/L)	167	219	482
25OHD (> 50 nmol/L)	< 15	5.2	12.5
X-ray knee	Fraying and splaying of the metaphyses characteristic of rickets	Fraying and splaying of the metaphyses characteristic of rickets	Fraying and splaying of the metaphyses characteristic of rickets
ECG - QTc (< 450 ms)	547	485	455
Echocardiography	Dilated CMP	Dilated CMP	Dilated CMP
LV dimension in diastole (Z-score)	+4.6	+ 5.5	+ 6.5
LV EF (range: 55–70%)	25–30%	29%	25%
LV FS (range: 29–40%)	7%	7%	15%
Function	Global hypokinesia	Global hypokinesia	Global hypokinesia
Mitral regurgitation (MR)	Severe MR	Moderate MR	Severe MR
Structural defects	None	None	None
Maternal characteristics			
Multivitamin taken during pregnancy	Yes	Yes	No
Adjusted serum Calcium (2.2–2.6 mmol/L)^b^	2.42	2.31	2.25
PO_4_ (0.8–1.5 mmol/L)	1.18	1.29	1.1
ALP (25–105 IU/L)	77	161	86
PTH (15–65 ng/L)	54	91	87
25OHD (> 50 nmol/L)	33.1	19.8	24

Abbreviations: *ALP* alkaline phosphatase, *PO*_*4*_ phosphate, *PTH* parathyroid hormone, *25OHD* 25 hydroxy-vitamin D, *LV* left ventricle, *EF* ejection fraction, *FS* fractional shortening, *CMP* cardiomyopathy, *MR* mitral regurgitation. First column shows normal ranges in parentheses

^a^Initial PO4 was 3.51 mmol/L (post cardiac arrest) then continuously dropping to 0.47 mmol/L within 48 h. ^b^Serum calcium is adjusted for albumin by using the formula: Adjusted calcium = measured total calcium +  0.02 * (40 - [albumin in g/L])

**Fig. 2:**
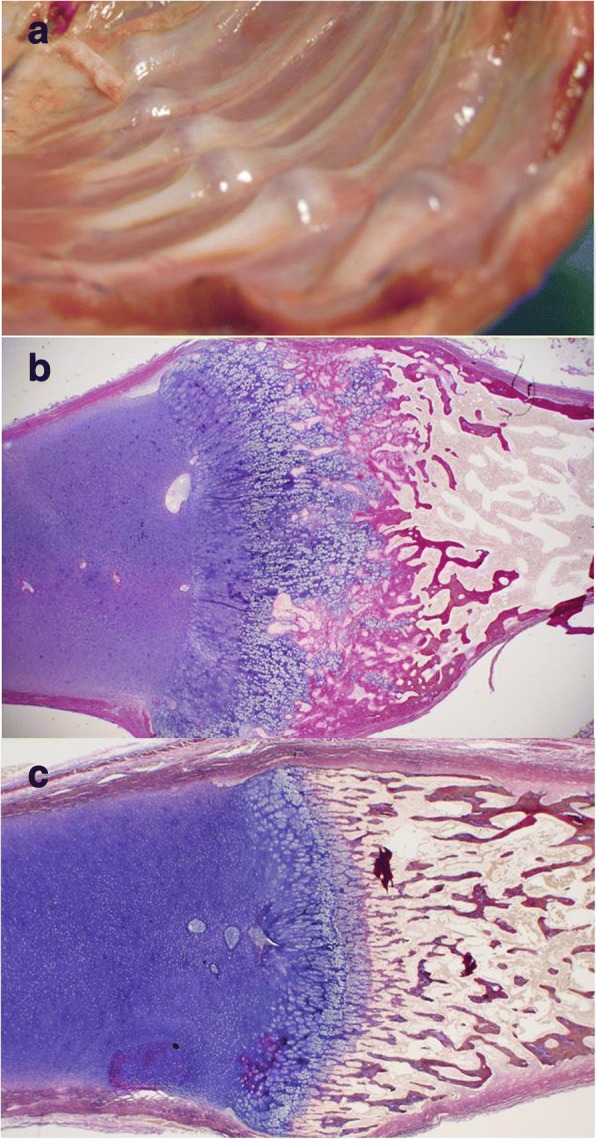
Post-mortem Findings**.** At post-mortem examination, Patient 1 had a rachitic rosary (**a**) and the rib growth plate showed extreme disarray (**b**, Elastica van Gieson staining). Normal growth plate in a 6 months-old control with normal 25OHD (**c**)

**Fig. 3:**
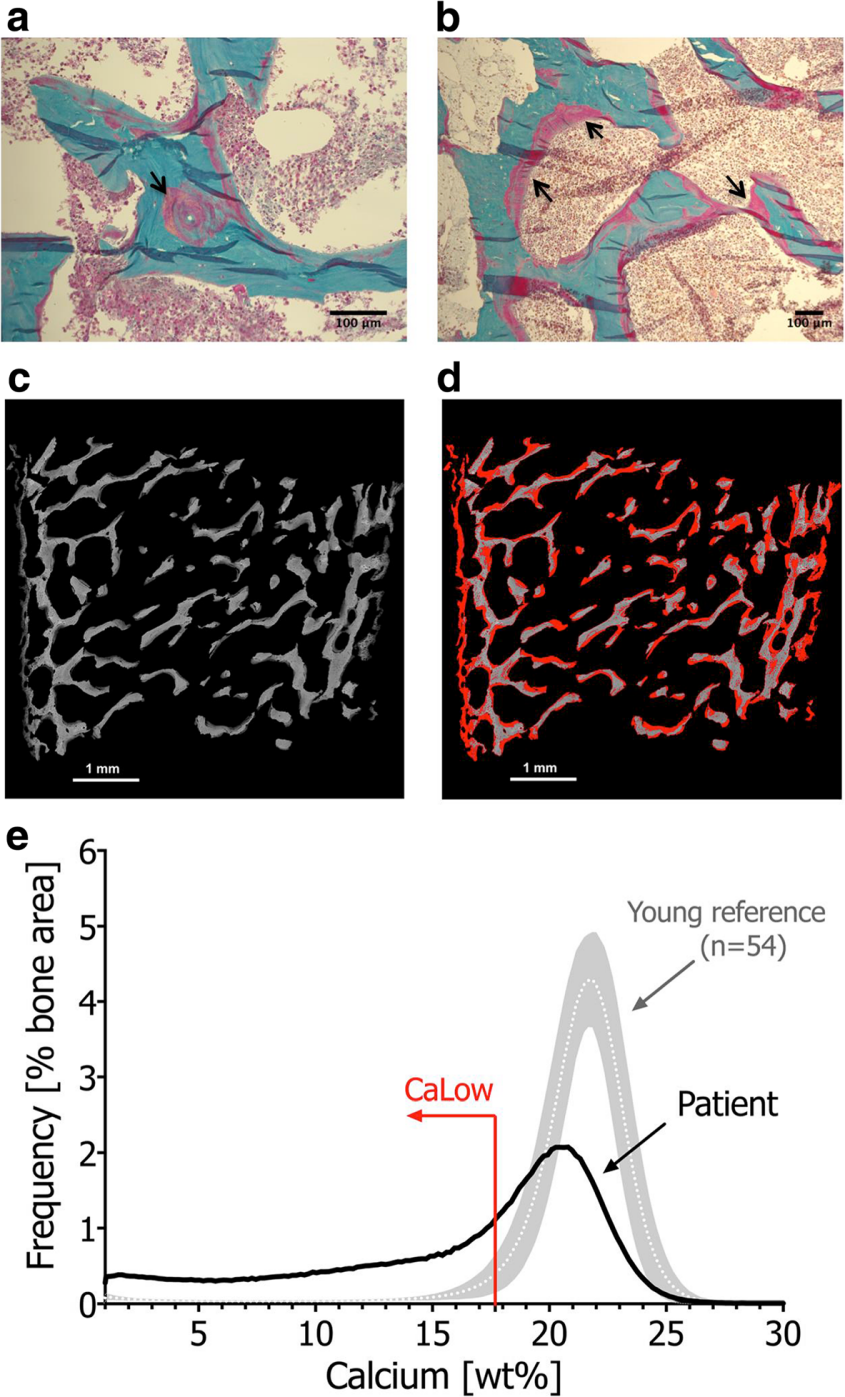
Histomorphometric and Quantitative Backscattered Electron Microscopic Analysis. Goldner’s Trichrome staining (light microscopy) of a post-mortem transiliac bone sample from Patient 1 (**a, b**) demonstrated broad seams of pink stained areas corresponding to non- or poorly mineralized matrix and regions with blurred pink-green transition (black arrows), next to mineralized matrix (green). Backscattered electron images of the complete bone sample surface (**c, d**) show low mineral content in dark grey, normal/high mineral content in bright grey and unmineralized matrix appears black (**c**). To demonstrate the massively increased primary mineralization, represented by areas mineralized below 17.68 wt% calcium, corresponding to the 5th percentile of the adult reference range (CaLow) [10], these areas were highlighted in red (**d**). The BMDD curve of patient 1 (**e**) was shifted towards lower mineral content, its width at half-maximum was broader (CaWidth + 55%) due to increased heterogeneity in mineralization, and the fraction of poorly mineralized matrix was markedly increased (CaLow + 640%). References from Fratzl-Zelman et al. [36]

Post-mortem examination confirmed severe nutritional rickets with rachitic rosary (enlarged rib growth plates) (Fig. 2a), craniotabes, soft ribs, dilated cardiomyopathy (heart weight 71 g [>95th centile], with multifocal myocardial necrosis) and massive ischaemic brain injury. Histological analysis of a 7th rib growth plate showed extreme disarray, widening and lengthening, with islands of hypertrophic chondrocytes reaching far into the primary spongiosa and mature bone, typical of rickets (Fig. 2b). Histomorphometric analysis of a transiliac bone sample identified severe osteomalacia with increased osteoid thickness (23.2 μm [normal 6.4 +/− 1.4]), osteoid surface/bone surface (76.3% [normal 24.9 +/− 10]) and osteoid volume/bone volume (40.5% [normal 2.4 +/− 1.22]). Specifically, osteoid thickness was + 262% and osteoid volume/bone volume + 1573% of normal reference values [9]. Since Goldner’s Trichrome staining does not discriminate well between non-mineralized and poorly mineralized matrix, we also performed quantitative backscattered electron imaging, which confirmed the extremely low bone mineralization density (Fig. 3).

The mother had received antenatal multivitamin supplementation and attended all post-natal child surveillance and vaccination appointments. She was not informed of the need for infant vitamin D supplementation. Mother (Table 1) and a 9-year old sibling had suboptimal 25OHD concentrations.

#### Patient 2

A 6-month old, partially breastfed and previously well Somali boy presented to the ED following respiratory arrest and seizure. He was found pale, floppy and not breathing while held by his sibling. Following emergency telephone advice, his mother, a nurse, commenced Cardio-pulmonary resuscitation (CPR) at home. Two minutes later he had a 2-min tonic-clonic seizure. With continued CPR, spontaneous breathing was established at 4 min. Paramedics found him drowsy with normal blood glucose. In the ED, he responded to pain, respiratory rate was 40/min, heart rate was 112/min with normal capillary refill. A grade 2/6 systolic ejection murmur was present. A venous blood gas was normal except for low ionised calcium (0.66 mmol/L). A chest radiograph showed cardiomegaly (Fig. 1c), and echocardiogram demonstrated a structurally normal heart with severely dilated left ventricle with reduced LVEF of 29%, FS of 7%, global hypokinesia and moderate mitral regurgitation, confirming hypocalcemic dilated cardiomyopathy. Diuretic and ACE (Angiotensin converting enzyme) inhibitor therapy was commenced. Nutritional rickets due to vitamin D deficiency was confirmed with knee radiographs (Fig. 1d), elevated serum ALP and PTH, and low 25OHD of < 5.2 nmol/L (Table 1). He received intravenous calcium and oral cholecalciferol (6000 IU daily). Alfacalcidol (1-hydroxycholecalciferol) was temporarily administered to improve calcium absorption. On day 3, following a switch from intravenous to oral calcium, he had another seizure with respiratory arrest in hospital, requiring mechanical ventilation and intensive care. Intravenous calcium was recommenced, and a head computed tomography was normal. He was extubated 24 h later and continued intravenous calcium for 5 more days. He was discharged home on day 17 and 3 months later showed slow recovery (LVEF 35%; FS 16%; Left ventricle diameter 42 mm [Z-score + 4.7], marked reduction in mitral regurgitation).

The mother had been provided with one bottle of vitamin D for the baby at birth but was not informed to continue supplementation, and adherence was not assessed. She (Table 1) and three of the infant’s four siblings (aged 3, 6, 7, 9 years) were vitamin D deficient, with elevated ALP and PTH.

#### Patient 3

A five-month old British Pakistani girl presented to ED with cough, difficulty in breathing and poor feeding. She was born at 35 weeks with a birth weight of 1.75 Kg (9th centile) and required admission to the neonatal unit for 6 days to establish oral feeding. At presentation, she was found to be pale, irritable, tachypnoeic and tachycardic. She had faltering growth (fall across ≥2 weight centiles) with a weight of 4.5 kg (< 0.4th centile) and length 58 cm (on 0.4th centile). She was diagnosed with bronchiolitis. Only the faltering growth triggered further investigations which identified hypocalcemia (1.96 mmol/L). Further evaluation of hypocalcemia revealed raised ALP and PTH, and low 25OHD of 12.5 nmol/L (Table 1) and rickets on knee radiograph (Fig. 1f). An echocardiogram performed in view of persistent tachycardia, systolic murmur and cardiomegaly on chest radiograph (Fig. 1e) revealed a structurally normal heart with a severely dilated left ventricle (LVEF of 25%, FS of 15%, global hypokinesia and severe mitral regurgitation), confirming hypocalcemic dilated cardiomyopathy. She was commenced on oral calcium supplements (500 mg/day in divided doses) and cholecalciferol (initially 3000 IU daily, later increased to 6000 IU daily) and transferred to our tertiary center for specialist cardiology care. She was commenced on diuretics and ACE inhibitors.

Nobody had informed mother of the need for vitamin D supplementation during pregnancy and infancy. Her 3 year old sibling had normal 25OHD levels, however mum was deficient with a raised PTH (Table 1).

### Summary of family investigations

Overall, five of the nine tested family members had vitamin D deficiency (25OHD < 30 nmol/L) and three had insufficiency (< 50 nmol/L). Six of the 9 members had elevated PTH and ALP levels (biochemical signs of osteomalacia) and received treatment doses of vitamin D. All family members were advised to commence lifelong supplementation.

## Discussion and conclusion

Several billion people worldwide belong to ethnic groups at high risk of vitamin D deficiency and complications from calcium deprivation. Their risk is largely determined by dark skin pigmentation, traditional diets, and cultural habits. These risk groups originate from South Asia, the Middle East or Africa, regions with abundant sunshine, but they also live as immigrants and residents in high-income countries, which are mostly geographically located in latitudes with limited ultraviolet B (UVB) light from sunshine which is essential for cutaneous vitamin D synthesis. Regions furthest away from the equator in both hemispheres do not get much UVB during winter and spring, resulting in a ‘vitamin D winter’; hence the further away from the equator, the longer the ‘vitamin D winter’. In cities like London or Berlin (51–52 degrees north) the ‘vitamin D winter’ lasts for 6 months (October to April), [11] hence it is no surprise that the infants we report presented in early spring. They have in common that their risk and need for supplementation went unrecognized, adherence with supplementation was not monitored, and that clinical symptoms were relatively silent until severe complications of hypocalcemia manifested. The extent of disease, only unveiled by X-rays, echocardiography, blood tests and post-mortem investigations, went unnoticed by parents and health care professionals alike. These cases were fully preventable and represent only the tip of the iceberg of widespread deficiency in risk groups. They expose a public health failure to address vitamin D deficiency as an important health problem with potentially devastating consequences.

The main body of the iceberg is widespread calcium deprivation from vitamin D and dietary calcium deficiencies [1, 2], which are most common in, but not exclusive to, ethnic risk groups. Vitamin D deficiency was present in 38% of native and 76% of migrant’s newborns [12] in Italy, and in 47% of female and 19% of male teenagers in Saudi Arabia [13]. A large, pooled European population study found 13% of people vitamin D deficient, with a 3–71 fold higher risk in ethnic subgroups with dark skin [14]. The debate around vitamin D deficiency has focused on bone health, but the full spectrum of clinical complications includes hypocalcemic seizures, tetany, skeletal myopathy, and life-threatening dilated cardiomyopathy. Infants and children are at greatest risk of hypocalcemic complications [11].

Dilated cardiomyopathy from prolonged hypocalcemia has a high mortality. All infants in small case series from India, the Middle East and England [7, 15–19] were aged 3 weeks to 12 months, had dark skin, and were not on vitamin D supplements. Of 16 infants from the London cohort, 12 needed inotropic support, 8 were ventilated, 6 had cardiac arrest, and 3 died [7]. Here we present hypocalcemic cardiomyopathy with clinically occult rickets as a cause of heart failure and sudden infant death despite apparently normal clinical development and growth in 2 of the 3 infants (Table 1). Different manifestations of calcium deprivation, such as hypocalcemic cardiomyopathy, prolonged QTc intervals, seizures and rickets often co-exist [11]; holistic assessment is therefore indicated. Incidental findings of rickets and cardiomyopathy in post-mortem studies in England also implicate a role of calcium deprivation in infant mortality [20, 21].

Hypocalcemic seizures in neonates and infants are often the first clinical signs of calcium deprivation, and the vast majority of reported cases are from high-risk ethnic groups in England [6] and elsewhere [8, 22–24]. Eighty-seven percent of children with hypocalcemic seizures in England were below 1 year of age and 27% were neonates, consistent with the well-known vertical transmission of vitamin D deficiency from mother to baby [6]. Consuming vitamin D-fortified formula milk does not protect against development of seizures [6] or rickets [25]. Hence, vitamin D supplementation needs to start at birth in all infants, independent of the mode of feeding [1, 2].

Elevated serum ALP and PTH serve as functional markers of calcium deprivation [26]. Rickets, a radiological diagnosis [1, 2], appears later in the disease course, once secondary hyperparathyroidism has caused hypophosphatemia. Hypophosphatemia inhibits apoptosis of hypertrophic chondrocytes, elongates the hypertrophic zone, widens and disrupts growth plate anatomy (Fig. 2) and mineralization of primary spongiosa (Fig. 3). Alongside the growth plate changes of rickets, secondary hyperparathyroidism also leads to excessive bone resorption, and the initiated remodelling cycles involve osteoblasts laying down poorly mineralizing matrix typical of osteomalacia (Fig. 3).

The incidence of nutritional rickets is rising globally [27] and hospitalisation from rickets is increasing in England [28]. The prevalence of histological osteomalacia in white European adults at post-mortem is as high as 25% [29]. In fact, clinically symptomatic individuals are not representative of the true burden of subclinical rickets and osteomalacia, as indicated by the biochemical results of family members presented here. The increasing prevalence of vitamin D deficiency globally mirrors the trends in nutritional rickets, with dark-skinned individuals at a highest risk [4].

In the wake of the ongoing European refugee influx, demographic population changes require robust public health programs to protect the most vulnerable. Universal vitamin D supplementation of all pregnant women and infants, as recommended by the Global Consensus [1, 2], has been the policy in most European countries. Factors significantly associated with good adherence in infants are universal supplementation independent of the mode of feeding, monitoring of supplementation during child surveillance visits, provision of information at birth and financial incentives [5]. The United Kingdom has the least effective policy implementation [5], 86% of parents are unaware of the existence of a rickets prevention program (infant vitamin D supplementation) [30], and monitoring of supplementation is non-existent. Similar to reports from Canada and New Zealand [31, 32], none of our cases had received vitamin D supplements despite the presence of national policies. The death and the morbidity of infants described here could have been prevented by vitamin D supplementation during pregnancy and infancy and monitoring of adherence alongside the vaccination program. Bolus oral administration of vitamin D to infants at routine vaccination appointments has also been a successful strategy to prevent deficiency [33].

A recent article [34] called into question unnecessarily high 25OHD targets and the existence of a pandemic of vitamin D deficiency. However, the article did not reflect a global, multi-ethnic perspective of the critical role of vitamin D in preventing serious, potentially fatal outcomes in children highlighted here. Supplementation with 600 IU and 400 IU of vitamin D has been recommended during pregnancy and infancy, respectively, not to reach high 25OHD targets, but to prevent rickets and the serious complications of hypocalcemia [1, 2].

In conclusion: Rickets was named the “English disease” during the industrial revolution, and has returned to England and other western countries through immigration of high-risk populations [35]. The morbidity and mortality from symptomatic vitamin D deficiency in infants is fully preventable. We call for renewed public health emphasis on strategies of vitamin D supplementation through food fortification and robust, accountable supplementation programs, with monitored adherence during routine prenatal and child surveillance visits.

## Abbreviations

25OHD: 25 hydroxyvitamin D
ACE: Angiotensin converting enzyme
ALP: Alkaline phosphatase
BAME: Black, Asian and Minority Ethnic
BMDD: Bone mineralization density distribution
CMP: Cardiomyopathy
CPR: Cardio pulmonary resuscitation
ED: Emergency department
FS: Fractional shortening
LVEF: Left ventricular ejection fraction
MR: Mitral regurgitation
MRI: Magnetic resonance imaging
PTH: Parathyroid hormone
UVB: Ultra violet B
